# Dissection of a locus on mouse chromosome 5 reveals arthritis promoting and inhibitory genes

**DOI:** 10.1186/ar2597

**Published:** 2009-01-20

**Authors:** Therese Lindvall, Jenny Karlsson, Rikard Holmdahl, Åsa Andersson

**Affiliations:** 1 Department of Experimental Medical Science, Unit for Medical Inflammation Research, BMC I11, Lund University, S-221 84 Lund, Sweden; 2 Department of Pharmacology and Pharmacotherapy, Faculty of Pharmaceutical Sciences, Copenhagen University, Universitetsparken 2, DK-2100 Copenhagen Ø, Denmark; 3 Current address: Pathology and Laboratory Medicine, University of California Los Angeles, 675 S. Charles E. Young Drive, Los Angeles, CA 90095, USA

## Abstract

**Introduction:**

In a cross between two mouse strains, the susceptible B10.RIII (H-2^r^) and resistant RIIIS/J (H-2^r^) strains, a locus on mouse chromosome 5 (*Eae39*) was previously shown to control experimental autoimmune encephalomyelitis (EAE). Recently, quantitative trait loci (QTL), linked to disease in different experimental arthritis models, were mapped to this region. The aim of the present study was to investigate whether genes within *Eae39*, in addition to EAE, control development of collagen-induced arthritis (CIA).

**Methods:**

CIA, induced by immunisation with bovine type II collagen, was studied in *Eae39 *congenic and sub-interval congenic mice. Antibody titres were investigated with ELISA. Gene-typing was performed by micro-satellite mapping and statistics was calculated by standard methods.

**Results:**

Experiments of CIA in *Eae39 *congenic- and sub-interval congenic mice, carrying RIIIS/J genes on the B10.RIII genetic background, revealed three loci within *Eae39 *that control disease and anti-collagen antibody titres. Two of the loci promoted disease and the third locus was protected against CIA development. By further breeding of mice with small congenic fragments, we identified a 3.2 mega base pair (Mbp) interval that regulates disease.

**Conclusions:**

Disease-promoting and disease-protecting genes within the *Eae39 *locus on mouse chromosome 5 control susceptibility to CIA. A disease-protecting locus in the telomeric part of *Eae39 *results in lower anti-collagen antibody responses. The study shows the importance of breeding sub-congenic mouse strains to reveal genetic effects on complex diseases.

## Introduction

Rheumatoid arthritis (RA) and multiple sclerosis (MS) are complex inflammatory autoimmune disorders in which genetic and environmental factors contribute to disease development [1]. RA is characterised by peripheral joint inflammation, cartilage and bone destruction and, subsequently, joint deformation. In MS, the myelin and axons are affected by inflammation within the CNS often leading to severe neurological dysfunction. The disease-causing mechanisms remain unknown, although it is known that the aetiology is dependent on multiple genetic and environmental factors. To date, only a few genes have been associated with susceptibility to RA [2-4] and MS [5,6].

The most commonly used animal model for RA is collagen-induced arthritis (CIA) [7]. The B10.RIII (H-2^r^) mouse strain develops poly-arthritis after immunisation with bovine type II collagen, whereas the RIIIS/J mouse strain, having the same major histocompatibility complex (MHC) haplotype (H-2^r^), is resistant to poly-arthritis development. Induction of CIA is dependent on genes within the MHC, but as previously shown in crosses between B10.RIII and RIIIS/J mice, non-MHC genes also play an important role in susceptibility to disease [8-10].

Experimental autoimmune encephalomyelitis (EAE) is an inflammatory demyelinating disease of the central nervous system (CNS), widely used as an animal model for MS. The B10.RIII strain is susceptible to EAE induced by the myelin basic protein (MBP) peptide 89–101 [11]. From studies of crosses between B10.RIII and RIIIS/J (resistant to EAE development), a number of non-MHC quantitative trait loci (QTLs), linked to EAE susceptibility, have been reported [12-14]. In one study, the *Eae39 *locus on mouse chromosome 5 was linked to acute EAE [13]. The inheritance pattern showed that RIIIS/J genes were dominantly protective. The *Eae39 *locus is the only QTL linked to EAE on mouse chromosome 5, but six QTLs linked to disease in arthritis models have been identified on this chromosome: *Cia13*, *Cia14 *and *Cia27 *for CIA [15,16], *Pgia16 *for proteoglycan-induced arthritis [17], and *Bbaa3 *and *Bbaa2 *for *Borrelia burgdorferi*-associated arthritis [18].

The *Eae39 *locus was identified as a genetic region of about 30 mega base pairs (Mbp). In order to further investigate the genetic control of disease in the B10.RIII/RIIIS/J model, we have studied CIA in *Eae39 *and *Eae39 *sub-interval congenic mice. We observed three different inheritance patterns associated with arthritis development, which argues that there are at least three genes in *Eae39 *that are important for the development of inflammatory disease. Two of the loci, located within a distance of a few Mbp, contain genes that, depending on the allele, either protect from or promote disease. This suggests a balancing effect by closely located genes on disease susceptibility that is revealed when QTLs are split into smaller fragments.

## Materials and methods

### Animals

**Figure 1:**
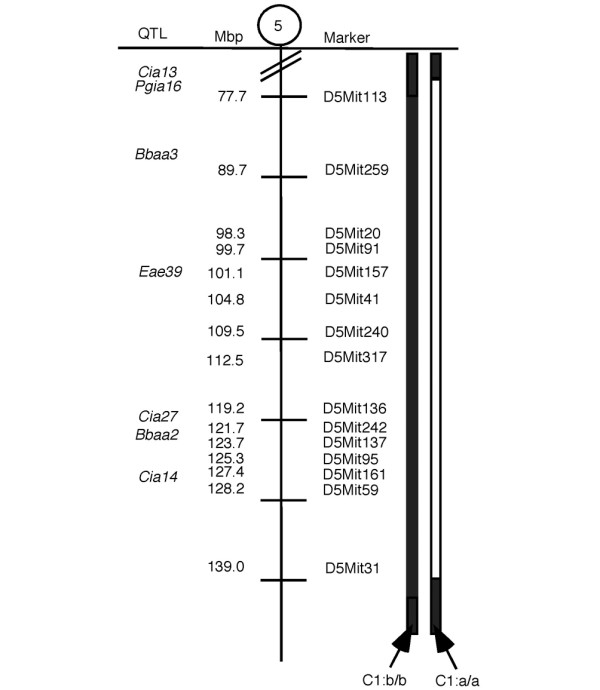
The *Eae39 *locus on mouse chromosome 5. The C1 congenic fragment is derived from RIIIS/J and bred on to the B10.RIII background by marker selected back-crossing. Black = two B10.RIII alleles; white = two RIIIS/J alleles. Mbp = mega base pairs (positions according to Ensembl release 49).

C57Bl/10.RIII (B10.RIII) were originally provided by J. Klein (Tübingen, Germany), and kept in the breeding colony at the Department of Medical Inflammation Research, Lund University. RIIIS/J animals were purchased from The Jackson Laboratory (Bar Harbor, ME). The *Eae39 *congenic mice (C1, Figure 1) were produced by marker selected backcrossing of the RIIIS/J (donor) mice to the B10.RIII (recipient) strain. All experiments were approved by the local ethical authorities in Malmö-Lund, Sweden (permit numbers: M70-04, M75-04, M107-07 and M109-07).

### Induction and evaluation of collagen-induced arthritis

Bovine type II collagen was prepared from calf nasal cartilage by pepsin digestion and was purified as previously described [19]. CIA was induced by intra-dermal immunisation at the base of the mouse's tail with 100 μg bovine CII emulsified in incomplete Freund's adjuvant (IFA) (Difco, Detroit, MI, USA). The mice were boosted 35 days later with 50 μg bovine CII emulsified in IFA. The mice, ranging in age between 10 and 24 weeks, were all immunised the same day. Clinical disease was monitored once or twice a week according to a scoring system based on the number of inflamed joints. Each inflamed toe or knuckle was given a score of one and an inflamed wrist or ankle was given five points. Each mouse could in total get 15 points per limb and a maximum score of 60. The area under the curve (AUC) was calculated as the sum of scores for each individual mouse during a defined test period. The mean maximum score, representing disease severity, was calculated as the mean of the maximum score of all sick mice in the respective groups.

### Antibody measurement

Blood was collected on day 14, 21 or day 54 after immunisation. Sera were prepared and stored at -20°C until assayed. ELISA was used to determine levels of antibodies against collagen type II. Plates (Nunc maxisorp, Roskilde, Denmark) were coated with bovine CII (10 μg/ml) in PBS (pH 9) and blocked with 1% BSA. Immunoglobulin (Ig) M, IgG1, IgG2c, IgG3 and total Ig levels were measured using biotinylated secondary antibodies: goat anti-mouse IgM (No. 1020-08); IgG1 (No.1070-08); IgG2c (No. 1079-04); IgG3 (No. 1100-08); and total Ig (No. 1010-08) (Southern Biotechnologies Associates, Birmingham, AL, USA). Binding of biotinylated antibodies was revealed by Extravidin Peroxidase (No. E-2886) (Sigma-Aldrich, St Louis, MO, USA). Plates were developed with ABTS: 2,2'-Azino-di-[3-ethylbenzthiazoline sulfonate (6)] diammonium salt (Roche, Mannheim, Germany). Pooled sera were used as a standard and the antibody levels were measured as arbitrary concentrations.

### Genotyping and linkage analysis

Genomic DNA was isolated from toe or tail biopsies. The biopsies were dissolved in 500 μl of 50 mM sodium hydroxide for one to two hours at 95°C, and subsequently neutralised with 100 μl 1 mM Tris-HCl (pH 8). To perform a standard 10 μl PCR, 1 μl of the solution was used. The PCR products were analysed on a MegaBACE DNA analysis system 1000 (Amersham Pharmacia Biotech, Little Chalfont, UK), according to the manufacturer's protocol. Fifteen informative fluorescence-labelled micro-satellite markers (Interactiva Biotechnologie, Ulm, Germany and MWG Biotech, Ebersberg, Germany) were used to genotype the *Eae39 *congenic fragment. Linkage analysis and permutation tests were conducted as previously described [13]. Sub-congenic mice were genotyped with additional micro-satellite markers, where some markers are made in-house: D5acacbhm4 (114.42 Mbp, forward primer (FP) 5'-CCCTGTAGAAGACTGGGAATTG-3, reverse primer (RP) 5'-TCCAGGACAGTCAGGGCTAC-3'), D5taokhm12 (117.53 Mbp, FP: 5'-TCAGGGCTCCATGCACTT-3', RP: 5'-CACAAGTGGCTCTCAGTGCT-3), D5sdshm18 (120.74 Mbp, FP: 5'-GGGGAACACAAGGAGTTTGA-3', RP: 5'-ATTCAAGGGCATGTGTGTGA-3').

## Results

### *Eae39* controls collagen-induced arthritis

The *Eae39 *locus on mouse chromosome 5 (Figure 1) was previously described in a genetic linkage analysis based on a backcross between the RIIIS/J and B10.RIII strains [13]. The confidence interval for *Eae39 *extended from the micro-satellite marker D5Mit259 (90 Mbp) to D5Mit136 (119 Mbp). This locus was shown to control incidence of acute EAE in male mice, with a dominant effect of RIIIS/J alleles on protection from disease development.

**Figure 2:**
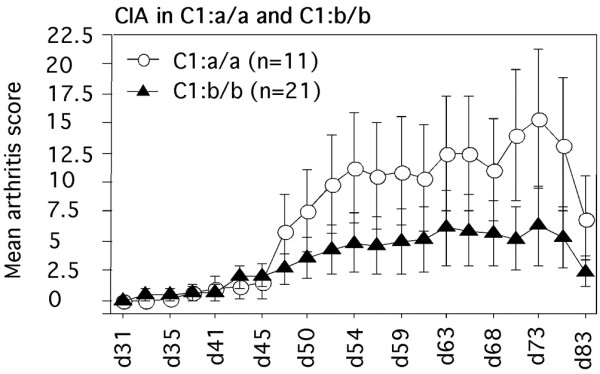
Collagen-induced arthritis (CIA) development in mice with the C1 congenic fragment. a/a (area under the curve (AUC) (d50-73) = 125 ± 50, incidence = 64%), b/b (AUC (d50-73) = 57 ± 29, incidence = 24%), (AUC (d50-73) p = 0.0379, Mann-Whitney U test, incidence p = 0.0271, Chi squared test).

To further investigate the *Eae39 *locus, a 65 Mbp RIIIS/J fragment was bred into the B10.RIII genome to establish a BR. *Eae39*^*RIIIS/J*^congenic strain (Figure 1). This region contains a number of QTLs linked to the development of disease in experimental models for arthritis, and we wanted to investigate whether *Eae39 *controls the susceptibility to CIA in the B10.RIII and RIIIS/J strain combination. Thus, *Eae39 *congenic mice were immunised with bovine collagen type II in IFA. As shown in Figure 2 and Tables 1 and 2, genes within *Eae39 *control CIA. Figure 2 shows the development of disease in mice with the congenic *Eae39 *fragment shown in Figure 1. Mice with the C1 congenic fragment had higher incidence of disease (64%) and higher accumulated arthritis score (AUC (d50-73) = 125 ± 50) compared with the non-congenic littermates (incidence of disease 24%, AUC (d50-73) = 57 ± 29). (Incidence, p = 0.0271, Chi squared test; AUC (d50-73), p = 0.0379, Mann–Whitney U test).

**Table 1:** Incidence and mean maximum score of collagen-induced arthritis (CIA) in *Eae39 *congenic mice^a^

Phenotype	Marker	Mbp^b^	Group	a/a^c^	a/b	b/b	p-value^d^
Incidence	D5Mit113	77.68	Total	10/14 (71%)	7/12 (58%)	8/42 (19%)	0.0005
			Males	3/5 (60%)	4/9 (44%)	2/21 (10%)	0.0222
			Females	7/9 (78%)	3/3 (100%)	6/21 (29%)	0.0082
							
	D5Mit136	119.18	Total	11/15 (73%)	3/19 (16%)	11/34 (32%)	0.0019
			Males	3/5 (60%)	0/10 (0%)	6/20 (30%)	0.0345
			Females	8/10 (80%)	3/9 (33%)	5/14 (36%)	0.0573
							
Mean max score^e^	D5Mit113	77.68	Total	23 ± 5	21 ± 5	21 ± 7	0.7766
			Males	31 ± 15	20 ± 10	14 ± 2	0.3280
			Females	20 ± 5	22 ± 10	24 ± 9	0.9604
							
	D5Mit136	119.18	Total	21 ± 5	6 ± 4	27 ± 15	0.1034
			Males	31 ± 15		18 ± 3	0.1948
			Females	18 ± 5	6 ± 4	37 ± 6	0.0317

^a^Shows the mean incidence and the mean maximum score of mice with the respective genotypes on markers D5Mit113 and D5Mit136. Calculations were made on all mice in Figures 1 and 3 (a to l), and littermate controls. The sub-interval congenic mice were generated by intercrossing the C1 congenic mice (Figure 1).

^b^Mbp = mega base pairs. The Mbp position is according to Ensembl release 49.

^c^a/a = homozygous RIIIS/J alleles; b/b = homozygous B10.RIII alleles; a/b = heterozygous.

^d^Statistics for incidence was calculated with Chi squared test. Statistics for severity was calculated with Kruskal-Wallis test and Mann-Whitney U test.

^e^Mean of the maximum score for all affected mice in Figures 1 and 3.

**Table 2:** CIA severity in *Eae39 *congenic mice

		AUC (d50-73)^a^			
		
Marker	Mbp^b^	a/a^c ^(n)	a/b (n)	b/b (n)	p-value^d^
D5Mit113	77.68	126 ± 41 (14)	87 ± 35 (12)	30 ± 15 (42)	0.0006
D5Mit157	101.06	118 ± 39 (15)	41 ± 17 (27)	46 ± 24 (26)	0.0071
D5Mit240	109.52	118 ± 39 (15)	33 ± 16 (27)	55 ± 25 (26)	0.0075
D5Mit136	119.18	118 ± 39 (15)	4 ± 4 (19)	66 ± 22 (34)	0.0023
D5Mit367	120.31	111 ± 37 (16)	5 ± 4 (17)	66 ± 22 (34)	0.0077
D5Mit137	123.73	107 ± 44 (13)	21 ± 13 (22)	68 ± 22 (33)	0.1014
D5Mit95	125.31	99 ± 41 (14)	22 ± 14 (21)	68 ± 22 (33)	0.1799
D5Mit161	127.40	106 ± 44 (13)	24 ± 14 (35)	64 ± 21 (20)	0.2488
D5Mit59	128.20	99 ± 41 (14)	25 ± 15 (19)	66 ± 22 (34)	0.2322
D5Mit31	139.00	125 ± 50 (11)	47 ± 34 (8)	48 ± 16 (49)	0.0423

^a^Area under curve (AUC) is the mean ± standard error of the total sum of scores for mice with the respective genotypes (day 50 until day 73). All mice in Figures 1 and 3 (a to l), and littermate controls (b/b) are included in the calculations. The sub-interval congenic mice were generated by intercrossing the C1 congenic mice (Figures 1 and 2).

^b^Mbp = mega base pairs. The Mbp position is according to Ensembl release 49.

^c^a/a = homozygous RIIIS/J alleles; b/b = homozygous B10.RIII alleles; a/b = heterozygous.

^d^Statistics calculated with Kruskal-Wallis test.

**Figure 3:**
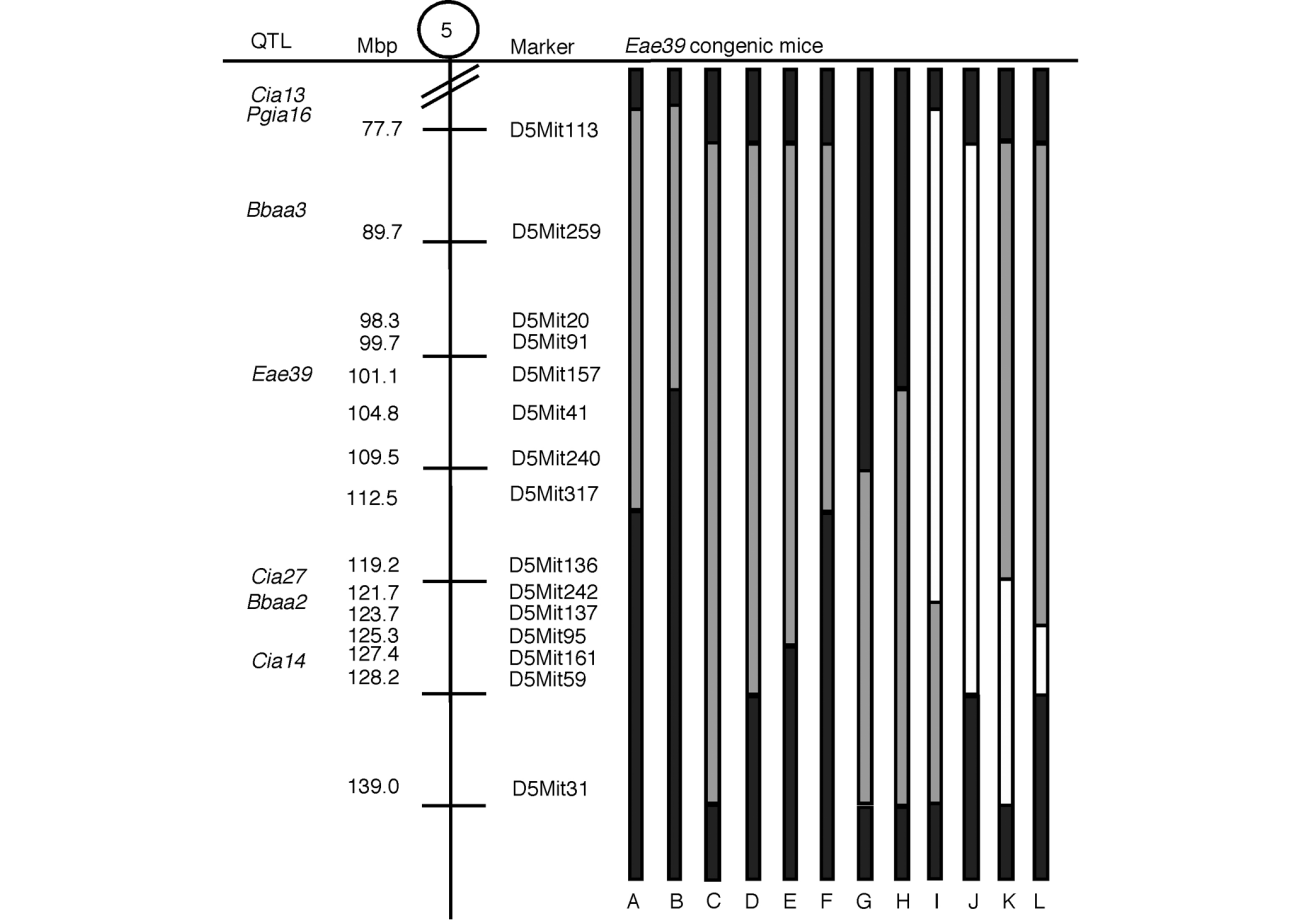
*Eae39 *sub-interval congenic mice. The sub-interval congenic mice were generated by intercrossing heterozygous C1 congenic mice. Black = two B10.RIII alleles; white = two RIIIS/J alleles; grey = heterozygous. Mbp = mega base pairs (positions according to Ensembl release 49).

Next, we intercrossed C1 heterozygous mice in order to get offspring with overlapping congenic fragments (Figure 3) to pinpoint smaller intervals within *Eae39 *linked to the disease phenotype. The mice were investigated for development of CIA and each individual was genotyped with markers spanning the congenic fragment. We observed that RIIIS/J alleles at marker D5Mit113 (78 Mbp) promoted disease incidence (Table 1). In contrast, one RIIIS/J allele at the marker D5Mit136, in the telomeric part of the fragment, protected against CIA development (Table 1). There was no difference in mean maximum score of the affected mice, except for females with RIIIS/J alleles at marker D5Mit136, which had significantly lower CIA scores compared with littermate controls (Table 1). This is in line with the male mice carrying heterozygous alleles at D5Mit136, where none of the mice developed arthritis.

In Table 2, correlation between the disease severity phenotype AUC, day 50 to 73 after immunisation, and genotype is shown. The AUC is the sum of scores for each individual mouse during a defined test period and describes the development of disease in terms of onset, duration and severity. In line with the disease incidence data (Table 1), RIIIS/J alleles at D5Mit113 promoted disease, whereas one RIIIS/J allele at about 120 Mbp (D5Mit136, D5Mit367) almost completely protected from CIA (Table 2). From these results we conclude that *Eae39 *harbors genes that, in addition to controlling EAE, are important for susceptibility to CIA, and that the region contains genes operating in different directions in the disease development.

In humans, women are more affected by RA than men. The sex influence on susceptibility to CIA is, however, normally the opposite in mice. In the first investigation of CIA development in mice with overlapping *Eae39 *sub-interval congenic fragments, we observed that female congenic mice had the same, or slightly higher, incidence of disease compared with male mice (Table 1). Severity of CIA (mean maximum score) was the same, except for females homozygous for B10.RIII alleles (b/b) at marker D5Mit136, in which the severity was higher (p < 0.05) compared with male mice (Table 1). From the original mapping experiment, *Eae39 *was linked to development of acute EAE in male mice [13]. For this reason, and in order to keep the number of mice used to a minimum, we decided to continue the present study with male mice only.

### The collagen type II antibody response is controlled by genes in the *Eae39* locus

**Figure 4:**
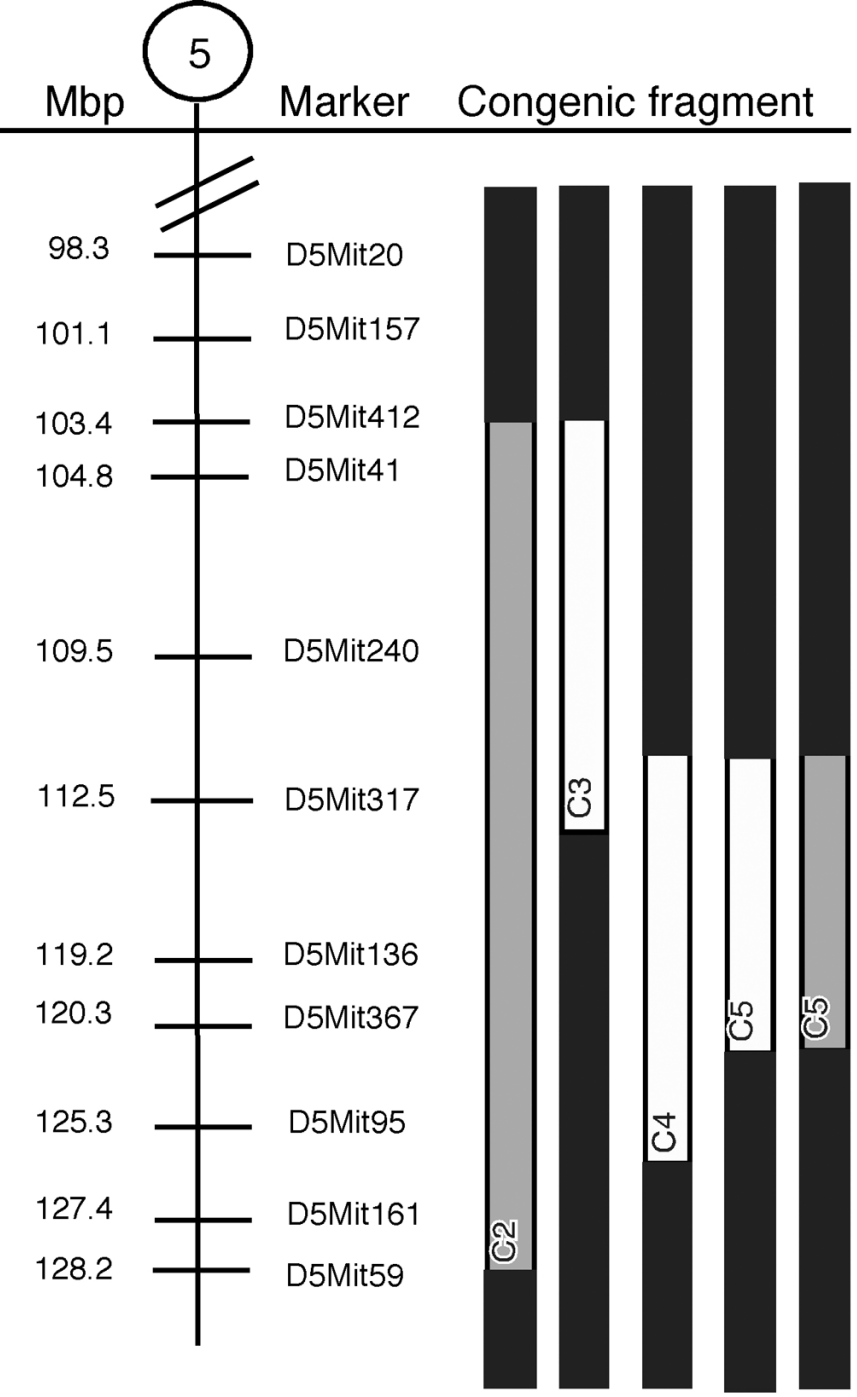
Schematic outline of congenic fragments in the *Eae39 *locus. C2 (D5Mit412 – D5Mit59); C3 (D5Mit412 – D5Mit317); C4 (D5Mit317 – D5Mit95); C5 (D5Mit317 – D5Mit367). The C3 and C4 fragments were generated by backcrossing the C2 fragment to the parental B10.RIII strain and subsequently intercrossing the offspring. The C5 fragment was generated by backcrossing the C4 fragment to the parental B10.RIII strain and subsequently intercrossing the offspring. Black = two B10.RIII alleles; white = two RIIIS/J alleles; grey = heterozygous.

In an F2 cross between the arthritis susceptible DBA/1J and the resistant FVB/N strains, it was recently shown that the *Cia27 *locus controls anti-collagen type II IgG2a antibody levels [16]. To investigate the corresponding region within the *Eae39 *locus for disease phenotypes, we produced mice with smaller, overlapping congenic fragments (C2 to C5) (Figure 4) and studied the anti-collagen antibody response after immunisation. The IgG1, IgG2c, IgG3 and IgM anti-collagen serum levels at day 14 after immunisation were significantly lower in mice with the C2 congenic fragment (Figure 4) compared with littermate controls (Table 3). By comparing antibody levels in mice with the C3 and C4 congenic fragments, we found that the anti-collagen type II serum titres of the IgG2c isotype were significantly lower in mice with the C4 fragment compared with littermates and to mice with the C3 fragment. Mice with the C5 fragment (spanning from D5Mit317 (112 Mbp) to D5Mit367 (120 Mbp)) had significantly lower IgG1, IgG2c, IgG3 and total Ig serum levels compared with littermate controls (Table 3). This confirms the effect on the antibody response observed with the C2 fragment and shows that genes in this region control antibody responses to type II collagen.

**Table 3:** Anti-collagen type II antibody responses in C2, C3, C4, and C5 congenic mice

Congenic fragment^a^	Day after immunisation	Antibody isotype	a/a^b^	a/b^c^	b/b^d^	p-value^e^
C2	14	IgG1		65^f ^± 7^g^	115 ± 13	0.0006
		IgG2c		80 ± 8	114 ± 12	0.0172
		IgG3		97 ± 12	176 ± 25	0.0017
		IgM		113 ± 9	171 ± 15	0.0003
						
C3	54	IgG1	187 ± 30		381 ± 118	0.2667
		IgG2c	1468 ± 405		987 ± 373	0.6634
		IgG3	238 ± 46		275 ± 62	0.2852
		IgM	102 ± 15		155 ± 57	0.4862
						
C4	54	IgG1	162 ± 57		272 ± 99	0.3465
		IgG2c	350 ± 73		797 ± 162	0.0280
		IgG3	176 ± 52		214 ± 86	0.4118
		IgM	81 ± 9		89 ± 17	>0.999
						
C5	14	IgG1	139 ± 20	258 ± 37	335 ± 63	0.0172
		IgG2c	240 ± 71	368 ± 73	1201 ± 409	0.0076
		IgG3	89 ± 18	142 ± 19	189 ± 24	0.0132
		IgM	306 ± 68	633 ± 98	875 ± 142	0.0041

^a^Congenic fragments according to Figure 4.

^b^Male mice with homozygous RIIIS/J alleles in C3 (n = 22), C4 (n = 12) and C5 (n = 10).

^c^Male mice with heterozygous alleles in C2 (n = 45) and C5 (n = 10).

^d^Male mice with homozygous B10.RIII alleles in C2 (n = 28), C3 (n = 9), C4 (n = 8) and C5 (n = 7).

^e^Statistics was calculated with Mann-Whitney U test and Kruskal-Wallis test.

^f^Arbitrary antibody concentration. Anti-collagen type II antibodies in non-immunised mice are not detectable.

^g^Mean ± standard error of the mean.

### Collagen-induced arthritis development and antibody responses to type II collagen in the C5, C6, C9, C10, and C11 congenic mice

**Figure 5:**
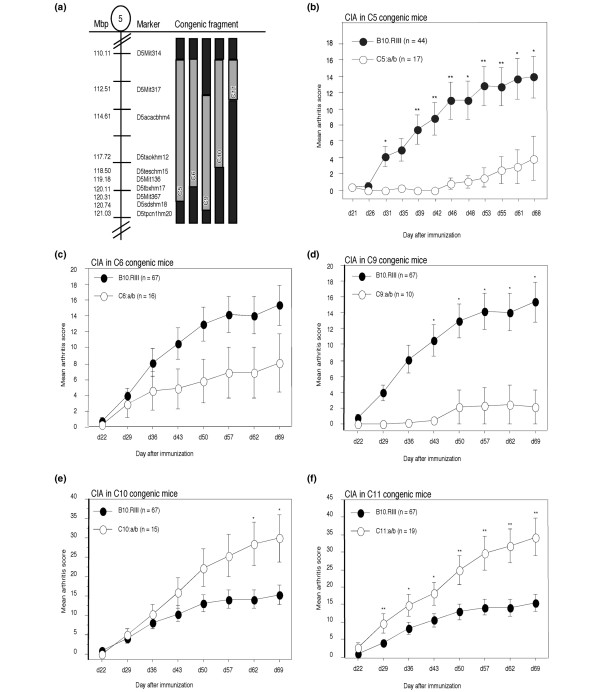
Collagen-induced arthritis (CIA) in *Eae39 *congenic mice. **(a) **A schematic outline of overlapping congenic fragments confined to the C5 interval. Black = two B10.RIII alleles; grey = heterozygous. **(b - f)** CIA development in C5, C6, C9, C10 and C11 congenic mice, and littermate controls. The littermate control group comprises all mice homozygous for B10.RIII alleles (b/b) from the breeding of the congenic mice. The different littermate control groups' data were pooled because they had similar disease progression. The C5 and C9 congenic fragment have been generated from C4 (Figure 4) by backcrossing to the B10.RIII parental strain and subsequently intercrossing the offspring. The C6, C10 and C11 were generated by backcrossing C5 congenic mice to the B10.RIII parental strain followed by intercrossing of the offspring. Stars indicate significant differences in mean arthritis score: * p < 0.05, ** p < 0.01.

Investigation of CIA development in C5 congenic mice showed that mice with one RIIISJ allele in this interval are protected from disease development compared with littermate controls (C5 congenics, incidence = 19%, mean maximum score = 24 ± 9; littermate controls, incidence = 50%, and mean maximum score = 31 ± 3; Table 4 and Figure 5b).

**Table 4:** CIA phenotypes in C5, C6, C9, C10, and C11 congenic mice

Phenotype^a^	B10.RIII^b^	C5	B10.RIII^c^	C6	C9	C10	C11
		
Incidence	22/44 (50%)	3/17 (19%)*	32/67 (48%)	6/16 (38%)	1/10 (10%)*	10/15 (67%)	14/19 (74%)*
Onset^d^	37 ± 2^e^	49 ± 3	34 ± 2	34 ± 4	36	32 ± 1.5	29 ± 1
Severity^f^	31 ± 3	24 ± 9	35 ± 3	24 ± 6	24	46 ± 3	48 ± 3*
AUC^g^	101 ± 20	13 ± 8*	80 ± 13	40 ± 19	10 ± 10*	137 ± 28	166 ± 27**

^a ^Values for incidence, onset, mean maximum score and area under the curve (AUC) are the mean phenotype values for the respective congenic mice. The congenic intervals are outlined in Figure 5a. Statistics was calculated with Chi squared test for incidence and Mann-Whitney U test for onset, severity and AUC. * p < 0.05, ** p < 0.01.

^b^B10.RIII = littermate controls for mice with the C5 congenic (a/b) fragment.

^c^B10.RIII = littermate controls for mice with the C6, C9, C10 and C11 congenic (a/b) fragment.

^d^The day for onset of disease.

^e^Mean ± standard error of the mean

^f^Severity is the mean of the maximum score of all affected mice in the respective group.

^g^AUC is the mean of the total sum of scores for mice in the respective group (day 21 to 69).

To further dissect the C5 region within the *Eae39 *locus, we bred congenic mice with overlapping fragments spanning the C5 region (C6, C9-C11) (Figure 5a). The new congenic mice were investigated for CIA and antibody responses to type II collagen. When splitting up the C5 fragment, we observed two different disease patterns. Mice with the C9 congenic fragment, which in contrast to C5 does not include the D5Mit317 marker, had a similar non-severe disease pattern to mice with the C5 fragment (Table 4, Figure 5d). Mice with the C6 fragment, covering the centromeric part but lacking the most telomeric part of the C5 fragment, developed more severe arthritis compared with mice with the C9 fragment (Table 4, Figures 5c,d). In mice with the C10 and C11 congenic fragments, a different pattern of disease development was observed because these mice were no longer protected from CIA, but instead developed more severe disease compared with littermate controls (Figures 5e and 5f, Table 4).

The anti-collagen type II antibody titre was not significantly lower in mice with the C6 and C9 fragments compared with the controls (Table 5). In the C10 and C11 congenic mice, the collagen type II antibody levels followed the disease course and the antibody concentrations were significantly higher compared with the littermate controls (Table 5).

**Table 5:** Anti-collagen type II antibody responses in C6, C9, C10 and C11 congenic mice

Antibody isotype	B10.RIII^a^	C6	C9	C10	C11
IgG1	2046^b ^± 276^c^	1567 ± 265	1354 ± 193	2520 ± 388*	2868 ± 576*
IgG2c	2337 ± 262	1754 ± 212	2031 ± 420	2859 ± 477	4096 ± 692**
IgG3	1873 ± 141	1364 ± 273	2144 ± 473	2483 ± 370	3021 ± 409**
IgTot	2705 ± 300	2038 ± 291	2439 ± 661	4130 ± 551**	3696 ± 543**

^a^B10.RIII = littermate controls (n = 64) for mice with the different congenic fragments. The congenic intervals are outlined in Figure 5a. C6 (n = 14), C9 (n = 9), C10 (n = 15), C11 (n = 17).

^b^Arbitrary antibody concentration in serum. Blood was collected day 21 after immunisation. Anti-collagen type II antibodies in non-immunized mice are not detectable.

^c^Mean ± standard error of the mean

Statistics was calculated with Mann-Whitney U test, * p < 0.05, **p < 0.01

In conclusion, when splitting up the C5 fragment into smaller intervals, we suggest a disease-controlling gene (or genes) close to the D5Mit317 marker in the upper part of the fragment. This part of C5 is shared with the disease promoting congenic fragments C10 and C11. Although not statistically significant for mice with the C6 fragment, the results from the C6 and C9 congenic mice suggest that a gene conferring protection against CIA development when one RIIIS/J allele is present, is located close to the D5Mit136 marker. In contrast to the C6 fragment, the C9 does not include the promoting gene/genes around the D5Mit317 marker, which could explain why the C9 congenic mice are more protected from disease development. Another possibility would be that there is another protecting gene close to the D5Mit367 marker, which is not present in the C6 fragment.

## Discussion

This study demonstrates that genes within the *Eae39 *on mouse chromosome 5 control development of CIA, and that this locus contains sub-loci that balance out each other in susceptibility to disease. By subdividing the original locus into smaller congenic intervals, we observe stronger effects on the disease phenotype in either direction. The original locus was defined in EAE, but here we show that *Eae39 *additionally controls CIA. Several QTLs for disease development in arthritis models have been mapped to this region: CIA (*Cia13*, *Cia14 *and *Cia27*) [15,16], proteoglycan-induced arthritis (*Pgia16*) [17] and *Borrelia burgdorferi*-associated arthritis (*Bbaa3 *and *Bbaa2*) [18]. The homologous regions in rats and humans have been linked to EAE development [20], pristane-induced arthritis [21], CIA [22] and RA [23,24], MS [25-27] and type 1 diabetes, respectively [28]. This suggests a shared genetic pathway in autoimmune diseases that is controlled by genes in this region.

**Figure 6:**
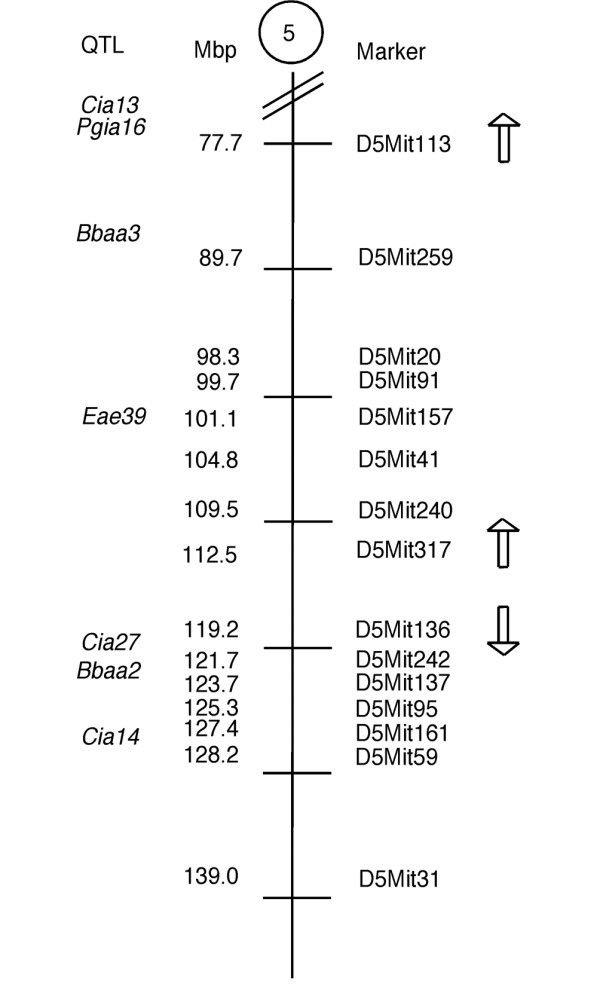
Collagen-induced arthritis (CIA) promoting- and protecting sub-loci within *Eae39*. The arrows indicate whether RIIIS/J alleles in this region enhanced or suppressed disease.

The *Eae39 *locus was previously identified in a backcross between the B10.RIII and RIIIS/J mouse strains and was shown to control acute EAE in male mice. The inheritance pattern showed that one RIIIS/J allele conferred protection from EAE [13]. We, and others, have previously demonstrated that loci linked to the development of polygenic diseases can consist of several sub-QTLs, operating in an additive fashion or in different directions in the control of the disease trait [9,10,29-32]. In the present study, we suggest that the original *Eae39 *locus harbors at least three genes that are involved in disease development (Figure 6). This could explain why the *Eae39 *locus was not found in previous EAE and CIA experiments with B10.RIII/RIIIS/J crosses, where the number of mice did not allow for the density of genetic recombinations needed to reveal a disease protecting or enhancing locus [8,14]. Mice with a small heterozygous *Eae39 *congenic fragment in the telomeric part of *Eae39 *were protected from disease. In contrast, homozygous RIIIS/J alleles in the complete *Eae39 *region or one RIIIS/J allele at the D5Mit113 (77.7 Mbp) marker, in the centromeric part of the fragment, promoted disease. This supports a complex inheritance pattern where RIIIS/J alleles in the centromeric part of *Eae39 *promote disease, whereas one RIIIS/J allele in the telomeric part of *Eae39 *protects against disease. The data could be explained by a strong dominant disease-promoting RIIIS/J gene close to D5Mit113, which overcomes the effect of the protecting RIIIS/J alleles in the telomeric part of the fragment. We have previously reported a similar inheritance effect between two QTLs, *Cia26 *and *Cia30*, within the *Eae2 *locus on mouse chromosome 15 [10].

Splitting up the disease protecting C5 fragment (Figure 5) into smaller congenic intervals, revealed opposing effects on CIA development. RIIIS/J alleles in the upper part of C5 (110.1 to 114.6 Mbp) strongly enhanced the disease, whereas mice carrying congenic fragments including the D5Mit136 marker (119.8 Mbp) were protected from disease development. The observation that the C5 fragment, sharing the disease-promoting parts with the C10 and C11 fragments, is protective, could be explained by a gene close to D5Mit136 that has a stronger effect on disease compared with the disease promoting gene located close to D5Mit317. The length of the protective region is 3.2 Mbp (117.7 to 121.0 Mbp). Except for the nitric oxide synthase 1 (*Nos1*), this interval contains no genes known to be directly involved in inflammation, but includes genes important in cell signalling, regulation (*Taok3*, *Wsb2*, *Rfc5*, *Ksr2*, *Tesc*) and development (*Tbx3*, *Tbx5*, *Lhx5*).

In addition to studies of CIA development in the *Eae39 *congenic mice, we investigated the antibody response to type II collagen after immunisation. We observed that the C5 congenic mice had lower antibody responses to collagen type II and were protected from disease development. In contrast, mice carrying the disease promoting C10 and C11 congenic fragments had enhanced anti-collagen antibody titres. This may suggest that the same gene(s) influence anti-collagen antibody titres together with the disease phenotype. Interestingly, Yu and colleagues [33] recently reported that the *Cia27 *locus on mouse chromosome 5 controls anti-collagen IgG2a antibody titres and the CIA disease phenotype. Although *Cia27 *was defined in an arthritis model with a different disease-inducing protocol and with mouse strains different from the strains used in the present study, it could be speculated that the same gene is operating in the two different models. *Cia27 *has been confined to 4.1 Mbp, and the peak marker is located at 120 Mbp, which corresponds to the genetic region found to control CIA disease phenotypes and anti-collagen type II antibody responses.

Male mice are normally more susceptible to CIA compared with female mice. In the present study, *Eae39 *sub-interval congenic female mice had as high an incidence of disease as male mice. Gender differences in susceptibility to CIA are believed to be dependent on hormones, genetic factors and behaviour [34]. We recently reported the identification of QTLs linked to CIA susceptibility in multiparous female mice [35], but this study did not reveal any linkage to mouse chromosome 5. Interestingly, the *Eae39 *locus includes two genes that are involved in the effects of oestrogen signalling; the G protein-coupled oestrogen receptor 1 (*Gper*, 139.9 Mbp) and oestrogen sulfotransferase (*Sult1e1*, 88.0 Mbp). The studies of smaller congenic fragments within *Eae39 *were performed with male mice and the gender susceptibility was not studied further. Investigations addressing any role for polymorphisms between B10.RIII and RIIIS/J in those genes would possibly contribute to the understanding of gender discrepancies in susceptibility to CIA.

This study demonstrates that breeding of mice with sub-congenic intervals, containing a limited number of genes, is informative in the dissection of QTLs defined in two-generation-crosses. Furthermore, it demonstrates that genes within the same disease pathways are located a close distance apart in the genome and possibly inherited together. Disease-protective polymorphisms have balancing effects, while a polymorphism in a different genetic context could increase the risk for disease.

## Conclusion

We have located a region in the telomeric part of *Eae39 *on mouse chromosome 5 that contains genes that control incidence and severity of CIA and serum levels of anti-collagen type II antibodies. In addition, we suggest that this region is influenced by a locus close to the marker D5Mit113 (77.7 Mbp), where B10.RIII alleles together with one RIIIS/J allele at marker D5Mit136 (119.2 Mbp) result in protection from disease. The disease-protecting region in the telomeric part of *Eae39 *is 3.2 Mbp and includes about 20 genes. Further studies will focus on the role of the genes within this sub-locus in the control of inflammatory disease- and sub-phenotypes.

## Abbreviations

AUC: area under curve; BSA: bovine serum albumin; CIA: collagen-induced arthritis; CNS: central nervous system; EAE: experimental autoimmune encephalomyelitis; ELISA: enzyme-linked immunosorbent assay; FP: front primer; IFA: incomplete Freund's adjuvant; Ig: immunoglobulin; Mbp: mega base pairs; MHC: major histocompatibility complex; MS: multiple sclerosis; PBS: phosphate-buffered saline; PCR: polymerase chain reaction; QTL: quantitative trait locus; RA: rheumatoid arthritis; RP: reverse primer.

## Competing interests

The authors declare that they have no competing interests.

## Authors' contributions

TL was responsible for the breeding of congenic mice, carried out the CIA and sub-phenotyping experiments, participated in the design of the study and drafted the manuscript. JK participated in the initial breeding of the congenic mice. RH participated in the design of the study and helped to draft the manuscript. ÅA participated in the design and coordination of the study, and helped to draft the manuscript.
